# Beverage Consumption Patterns among Infants and Young Children (0–47.9 Months): Data from the Feeding Infants and Toddlers Study, 2016

**DOI:** 10.3390/nu10070825

**Published:** 2018-06-26

**Authors:** Melissa C. Kay, Emily B. Welker, Emma F. Jacquier, Mary T. Story

**Affiliations:** 1 Duke Global Health Institute, Duke University, 310 Trent Dr, Durham, NC 27708, USA; emily.welker@duke.edu (E.B.W.); mary.story@duke.edu (M.T.S.); 2 Nestlé Research Center, Vers-Chez-les-Blanc, Route du Jorat 57, Case Postale 44, 1000 Lausanne-26, Switzerland; Emma.Jacquier@rd.nestle.com

**Keywords:** Feeding Infants and Toddlers Study, FITS 2016, beverage intake, breastfeeding, infants, toddlers, preschoolers, sugar-sweetened beverages, juice, water, milk

## Abstract

(1) Background: Data about early life beverage intake patterns is sparse. We describe beverage patterns among infants and young children from the Feeding Infants and Toddlers Study (FITS) 2016. (2) Methods: FITS 2016 is a cross-sectional survey of U.S. parents/caregivers of children 0–47.9 months (*n* = 3235). Food and beverage intakes were collected by 24-h dietary recalls to describe beverage consumption patterns including: a) prevalence of consumption, per capita and per consumer intake, b) contribution to intake of calories and key nutrients, and c) prevalence according to eating occasions. (3) Results: Breast milk and infant formula were commonly consumed among <12-month-olds. Among 12–23.9-month-olds, the most commonly consumed beverage was whole milk (67% consuming), followed by 100% juice (50% consuming). Plain drinking water was consumed by 70% of 12–23.9-month-olds and 78% of 24–47.9-month-olds. Among 12–47.9-month-olds, milks provided more energy and key nutrients than all other beverages. Across eating occasions, sugar-sweetened beverage (SSB) consumption, especially in the form of fruit-flavored drinks, was higher among 24–47.9 compared to 12–23.9-month-olds. Only 23–32% of ≥12-month-olds consumed milk or water at lunch or dinner. (4) Conclusions: Opportunities exist to improve beverage patterns. Future interventions may benefit from focusing on timely introduction of age-appropriate beverages and reducing consumption of SSBs.

## 1. Introduction

The period from birth to age 5 is a critical window for optimal growth and development [1]. During this early phase of life, dietary patterns begin to form and can influence later food and beverage preferences and health [2,3,4,5,6]. Beverages are a significant source of calories and nutrients during these first few years of life. In order to ensure a high-quality diet, several organizations have provided evidence-based guidance on the timing and types of beverages infants and young children should be consuming [7,8,9,10]. The American Academy of Pediatrics (AAP) recommends breast milk, or formula for those who cannot or choose not to breastfeed, for the first year of life and cow’s milk thereafter; plain drinking water can be introduced when the child shows developmental signs of readiness for solid food, which generally happens between 4–6 months [9]. Whole, unflavored cow’s milk is recommended for children 12–24 months of age; low-fat or fat-free milk is recommended starting at age 2, or for children at 12 months who have a family history of obesity or heart disease [7,11]. It is also recommended that 100% fruit juice be avoided until 12 months and then caregivers should limit 100% juice intake to no more than 4 fluid ounces per day for children aged 1–3 [12]. Sugar-sweetened beverages (SSBs) should be avoided entirely [9]. The 2015–2020 U.S. Dietary Guidelines for Americans (DGAs), intended for Americans aged 2 years and older, recommend that calories and nutrients be considered when choosing beverages. Further the DGAs recommend that calories from added sugars, which are in abundance in SSBs, be limited to less than 10% of daily calories [8]. For the purpose of this manuscript we refer to all drinks consumed under the collective term “beverages” including infant formula and breast milk, although they may be classified as “infant/baby foods” in food-group classification systems.

Currently, there is little data on beverage consumption patterns in the first years of life. Few studies have focused on the contribution of beverages to young children’s nutrient intake and the eating occasions during which specific beverages are consumed. The research that does exist demonstrates a misalignment between young children’s recommended and actual beverage and nutrient intake as they relate to the dietary guidance stated above. Overall, in the U.S. rates of exclusive breastfeeding are low, with less than a quarter of infants exclusively breastfed at 6 months and less than a third breastfed at 1 year; cow’s milk is being introduced before 12 months, and many children, including nearly a third of toddlers are consuming SSBs [13,14]. Beverage consumption patterns can have important implications on later childhood and adolescent dietary intakes [15]. For example, breastfeeding is associated with more positive dietary behaviors during childhood and longer durations of breastfeeding have been associated with more frequent fruit and vegetable consumption, less frequent SSB consumption, and reduced risk for obesity [16,17]. Additionally, infants who are given SSBs are more likely to consume them at age six [18]. Furthermore, Sonneville et al. found that children who consume 100% juice at 1 year of age may be more likely to consume higher amounts of 100% juice and SSBs in early and mid-childhood compared to children who do not drink any juice at al [19].

Assessing the types and amounts of beverages consumed by young children can also have significant impacts on nutrient adequacy [20,21]. Recent data show that among infants and toddlers, nutrients consumed in inadequate amounts include iron, fiber, calcium, vitamins D and E, and potassium, while saturated fat, sodium and added sugars are consumed in excess [8,22]. Milk is an important source of many key nutrients; however, early introduction of cow’s milk has been linked to iron deficiency [23]. Although 100% juice can be a good source of some nutrients, such as vitamin C, consuming 100% juice instead of whole fruit can displace fiber intake [8]. SSBs such as fruit-flavored drinks, soft drinks, and sports drinks contain few, if any nutrients, and may contribute to excess intake of added sugars and calories [7]. 

Few studies have assessed beverage intake among young children across eating occasions [24,25]. Previously, data from the Feeding Infants and Toddlers Study (FITS) demonstrated that consumption of beverages should be monitored as many young children were consuming 100% juice and SSBs at meals, especially at snack times [26,27,28]. Although there have been significant shifts in beverage patterns over the past several decades [14,29], no recent studies have focused on the eating occasions at which different types of beverages are consumed by infants and young children. This is critical information as it can inform targeted intervention efforts and practices to improve beverage patterns in young children. 

The purpose of this study is to use data from the FITS 2016 to describe the beverage consumption patterns of infants and young children (0–47.9 months), including (1) the prevalence of consumption, per capita and per consumer intake of beverages, (2) how beverages contribute to intake of calories and key nutrients, and (3) the prevalence of beverage consumption according to eating occasions.

## 2. Materials and Methods

### 2.1. Study Design and Participants

The FITS 2016 is a nationwide cross-sectional study of food, beverage and dietary supplement intake of young children aged 0–47.9 months living in the United States. This study builds on findings and methods in previous FITS studies conducted in 2008 and 2002 [30,31]. Extensive details about the FITS 2016 methodology have been published elsewhere [32]. Briefly, participant households were identified through stratified random sampling to achieve pre-determined sample sizes for 12 age groups and participation in the Special Supplemental Nutrition Program for Women, Infants and Children (WIC). Sampling weights were used to account for the probability of selection and response, and to reflect the U.S. population aged 0 to 47 months. A detailed overview of sampling frames is discussed by Anater and colleagues [32]. A total of 3235 infants and young children aged 0–47.9 months were included in the survey (see detailed demographic information in Supplementary Table S1). Instruments and methods from FITS 2008 were updated during the pilot-testing phase which lasted from January–May 2016. The full survey instrument consisted of: (1) a screening questionnaire to confirm respondent eligibility; (2) a recruitment questionnaire containing lifestyle and sociodemographic questions (completed by telephone or online); (3) a feeding practices questionnaire; and (4) one or two 24-h dietary recall interviews. All materials were available in both English and Spanish and were reviewed and approved by the Institutional Review Boards of RTI International; the University of Minnesota Nutrition Coordinating Center; and the Docking Institute of Public Affairs, Fort Hays State University. 

### 2.2. The 24-h Dietary Recall Procedure

Prior to completing the 24-h dietary recall, eligible respondents were mailed a packet of materials which contained written instructions, a food model booklet, ruler, liquid measuring cup and instructions on how to record foods and beverages that were fed by other adults/daycare. The 24-h recall was conducted over the phone, with the child’s primary caregiver, by trained interviewers, from University of Minnesota’s Nutrition Coordinating Center using the Nutrition Data System for Research (NDSR 2015, University of Minnesota, Minneapolis, MN, USA). Twenty-five percent of the respondents underwent a second 24-h dietary recall for the estimation of usual nutrient intake. More detailed information on the data collection procedures can be found in Anater and colleagues [32].

### 2.3. Data Analysis

All foods and beverages reported in the 24-h dietary recall were assigned to FITS food groups. The FITS food grouping system is largely identical to that used in FITS 2008 but was updated in order to be reflective of new foods and beverages and to bring the food groups into closer alignment with the “What We Eat in America” food group classification used in NHANES [33]. The method for coding breast milk consumption was consistent with that of past FITS surveys [31]. Pumped breast milk fed via a bottle was recorded as the volume consumed in a manner consistent with all beverages; however, breast milk consumed via breastfeeding was based on defined amounts by age for infants under 12 months, adjusted for the total volume of other milks consumed during the recall day, or by volume per feeding for children 12 months and older [31]. Based upon the FITS food group classification system beverage categories were identified as milks (i.e., flavored and unflavored cow’s milk, goat’s milk and plant-based milks), drinking water (tap, bottled or flavored), 100% fruit juice, and SSBs (i.e., fruit-flavored drinks, soft drinks, sports drinks, and sweetened tea and coffee); the FITS 2016 food group classification system groups breast milk and infant formula into a sub category called “baby milks”. Of note, consumption of milks includes milks added to cereal and used in recipes.

Using data from one 24-h dietary recall, the percentage of children consuming specific beverages on the day of the survey and the amounts of beverages consumed by those individuals (mean grams per consumer) was calculated. Per capita consumption data (mean grams) is also presented to provide an estimation of beverage intake behaviors at a population level, including consumers and non-consumers of each beverage. The energy provided from beverages (mean kcal per consumer and kcal per capita) has been reported elsewhere and is included in Supplementary Table S2 [34,35]. In addition, each beverage was ranked as a source of energy and nutrients among all foods and beverages consumed on the day of the survey with a specific focus on nutrients of concern [22]. Since most nutrients from beverages in the first year of life come from infant formula and breast milk, the analysis of nutrient intakes from beverages was conducted for children 12–47.9 months old only. Lastly, the percentage of children consuming different types of beverages at meal and snack occasions were calculated. Given that traditional meal patterns of breakfast, lunch, dinner and snacks do not become well established until around 12 months of age, beverage consumption at each meal and snack occasion was analyzed for 12–47.9-month-olds only [36].

## 3. Results

### 3.1. Sample Characteristics

Demographics of the FITS 2016 population have recently been published elsewhere [32], but briefly, the majority of children were non-Hispanic white (66%), 15% were non-Hispanic black and 15% were Hispanic. Almost all had a normal birth weight (91%); about a third of children (35%) received WIC benefits and roughly a quarter (27%) lived in a household that received SNAP benefits. Many (40%) of the respondents had at least a college degree and most (69%) were married. (Supplementary Table S1).

### 3.2. Beverage Consumption Patterns by Age Group

#### 3.2.1. Children 0–5.9 Month Old

Among 0–5.9-month-old infants, the majority consumed breast milk (54%) or infant formula (62%) on the day of the survey (Table 1). Few 0–5.9-month-olds consumed milks other than breast milk or formula (2%) and 100% juice (5%), and 10% consumed water. The average per capita intake of these beverages included 7 g of milks, coming mainly from whole milk; 4 g of 100% juice and 11 g of water. However, per consumer intake of milks was 367 g on the day of the survey, which is about 12 fluid ounces. Of those 0–5.9-month-olds consuming 100% juice on the day of the survey, they consumed an average of 86 g or about 3 fluid ounces. Those consuming water drank 115 g or roughly 4 fluid ounces. Few 0–5.9-month-olds consumed SSBs on the day of the survey (1%), but those that did consumed an average of 104 g.

#### 3.2.2. Children 6–11.9 Months Old

Among 6–11.9-month-olds, 39% consumed breast milk and 65% consumed infant formula on the day of the survey. About one in ten (11%) consumed milks with a mean per capita intake of 41 g. Among those consuming milks, average intake was 367 g. About one in four 6–11.9-month-olds (27%) drank 100% juice on the day of the survey, with an average of 42 g consumed per capita. Among consumers, an average of 152 g of 100% juice was consumed on the day of the survey, or about 5 fluid ounces. Prevalence of SSB consumption was 9% and the average amount consumed per capita was 11 g. The most commonly consumed SSB for this age group was fruit-flavored drinks, and among consumers of fruit-flavored drinks (7%), an average of 131 g were consumed on the day of the survey, nearly 4.5 fluid ounces. Almost half (46%) of 6–11.9-month-olds consumed water. Per capita intake was 69 g, and per consumer intake was 149 g, or almost 5 fluid ounces. 

#### 3.2.3. Children 12–23.9 Months Old

About one in ten (12%) 12–23.9-month-olds consumed breast milk (18% of 12–17.9 and 5% of 18–23.9-month-olds (data not shown)). Few (4%) consumed infant formula on the day of the survey. Most 12–23.9-month-olds (87%) consumed some variety of milk. Per capita intake was 401 g or nearly 13.5 fluid ounces, which is similar to the average grams per consumer of 460 g. The most commonly consumed variety of milk was whole milk (67% consuming) at 312 g per capita, followed by reduced fat milk (14% consuming) at 50 g per capita. Per consumer intakes of whole and reduced fat milk were 467 and 350 g, respectively. Half (50%) of 12–23.9-month-olds consumed 100% fruit juice at 112 g per capita or 3.8 fluid ounces. Among 100% juice consumers, children drank an average of 225 g or 7.6 fluid ounces on the day of the survey. Almost one in three (29%) 12–23.9-month-olds consumed SSBs on the day of the survey at 69 g per capita, about 2 fluid ounces. Average per consumer intake of SSBs in this age group was 237 g, nearly 8 fluid ounces. Fruit-flavored drinks were the most commonly consumed SSB in this age group and those children drinking fruit-flavored drinks on the day of the survey consumed an average of 239 g. Most (70%) 12–23.9-month-olds drank water on the day of the survey, consuming an average of 211 g per capita and 301 g per consumer or about 10 fluid ounces. 

#### 3.2.4. Children 24–47.9 Months Old

Few 24–47.9-month-olds consumed breast milk (4%) or infant formula (1%) with the majority (84%) consuming milks on the day of the survey. The most commonly consumed variety of milk was reduced fat milk (34% consuming), followed by whole milk (27% consuming), low-fat milk (23% consuming) and flavored milk (15% consuming). Of the 337 g (11.4 fluid ounces) per capita of milks consumed, 109 g came from whole milk, 104 from reduced fat milk, 93 from low-fat milk and 38 from flavored milk. Few (6%) 24–47.9-month-olds consumed non-fat milk. Children consuming milks on the day of the survey drank an average of 401 g or 13.6 fluid ounces. Almost half (47%) of 24–47.9-month-olds consumed 100% fruit juice at 113 g per capita (3.8 fluid ounces) and per consumer intake was 242 g or 8.2 fluid ounces. Nearly half (46%) of 24–47.9-month-olds consumed SSBs on the day of the survey. Per capita intake of SSBs was 136 g or 4.6 fluid ounces and per consumer intake was 299 g, or 10 fluid ounces. Fruit-flavored drinks were the most commonly consumed type of SSBs with 35% consuming them on the day of the survey. The average grams per consumer for fruit-flavored drinks was 263 g. Most (78%) 24–47.9-month-olds consumed water at 299 g per capita or 10 fluid ounces. Per consumer, children consumed an average of 383 g or 13 fluid ounces of water. 

### 3.3. Beverage Sources of Nutrients of Concern

Milks were the top contributor of any food or beverage for energy, saturated fat, calcium, vitamin D, vitamin A, and zinc among both 12–23.9 and 24–47.9-month-olds (Supplementary Table S3). Among 12–23.9-month-olds and 24–47.9-month-olds, milks were the 2nd and 4th highest contributor, respectively, of vitamin E. For 100% juice, it ranked as the 8th and 10th highest contributor of energy among 12–23.9 and 24–47.9-month-olds respectively, and also contributed some calcium, being 8th and 7th highest contributor, respectively. SSBs, largely fruit-flavored drinks, were the top contributor for added sugar intake among 12–47.9-month-olds of any foods and beverages consumed on the day of the survey, contributing 4 g per capita for 12–23.9-month-olds and 8 g per capita for 24–47.9-month-olds (data not shown). SSBs were also the 12th and 10th highest contributor of energy among 12–23.9 and 24–47.9-month-olds, respectively, contributing 3% and 4% to total energy intake (see Supplementary Table S2). 

### 3.4. Beverages Consumed at Eating Occasions

Given the low percentage of 12–47.9-month-olds consuming SSBs other than fruit-flavored drinks (Table 1), we present results for fruit-flavored drinks only across eating occasions. The most commonly consumed beverage at breakfast were milks, with a similar percent of children ages 12–23.9 (42%) and 24–47.9 (41%) months consuming them (Table 2). Compared to other beverage types, fruit-flavored drinks were the least commonly consumed beverage at breakfast. Water was the most commonly consumed beverage at lunch at 31% for both 12–23.9 and 24–47.9-month-olds followed closely by milks, then 100% juice. There was an increase in prevalence of fruit-flavored drink consumption at lunch between 12–23.9 and 24–47.9-month-olds (9% vs. 16%). At dinner, water and milks were the most commonly consumed beverages with similar prevalence across age groups at 31% and 30%, respectively for 12–23.9-month-olds and 32% and 31%, respectively for 24–47.9-month-olds. Beverage patterns differed among the two age groups at snack times with 57% of 12–23.9-month-olds consuming milks compared to only 36% of 24–47.9-month-olds. More 24–47.9-month-olds (59%) consumed water at snack compared to 12–23.9-month-olds (51%). Fruit-flavored drinks were consumed more frequently by both 12–23.9-month-olds and 24–47.9-month-olds at snack times (13% and 17%, respectively) than at breakfast (5% and 7%, respectively), lunch (9% and 16%, respectively) or dinner (9% and 11%, respectively).

## 4. Discussion

In this study, we used data from the FITS 2016 to describe beverage consumption patterns of infants and young children in the U.S. Overall, more children consumed infant formula as compared to breast milk with nearly half of infants under 12 months not consuming any breast milk at all. Some children consumed milks and 100% fruit juice before the age of 1 year. About half of 12–47.9-month-olds consumed 100% fruit juice; among consumers, children drank about 8 fluid ounces, which is more than recommended [12]. After age 24 months, some consumed whole and reduced fat milk, which are higher in saturated fat, while some did not have any milk at all; this is concerning given in our analysis milk was the top contributor among all foods and beverages for many nutrients of concern, including calcium, and vitamins D and A. While the DGAs’ recommendation of 2 cups per day includes all dairy foods (i.e., cheese and yogurt), milk is important given its contribution to other nutrient needs beyond calcium, such as potassium, vitamin D and protein [8]. Intakes of SSBs were high, nearly a third of 12–24.9-month-olds consumed them on the day of the survey and the prevalence rose to nearly half among 24–47.9-month-olds. The most commonly consumed type of SSBs was fruit-flavored drinks, which were most often consumed at lunch and snack times. Among consumers between 12 and 47.9 months of age the average intake of fruit-flavored drinks was approximately 8 to 9 fluid ounces/day. Overall, current beverage patterns of infants and young children are not in alignment with current dietary recommendations.

For the first year of life, nutrition recommendations emphasize consumption of breast milk, and when not possible, infant formula. Similar to national reports of breastfeeding rates, a little over half (53%) of 0–5.9-month-olds consumed breast milk on the day of the survey [37]. These results represent a higher rate compared to the 42% who consumed breast milk in both FITS 2008 and NHANES 2009–2014 [38,39]. Nearly 40% of 6–11.9-month-olds consumed breast milk, which is higher compared to FITS 2008 (33%) and NHANES 2009–2014 (26%). Interestingly, using FITS 2016 data, Roess et al. (in publication) reported 25% of toddlers aged 12–14.9 months consumed breast milk on the day of the survey, compared to 14% in FITS 2008 [34,38]. In fact, it is evident from FITS 2016 data that breastmilk consumption was more prevalent than infant formula consumption among 12–14.9-month-olds which is encouraging news [34]. These are promising results, perhaps reflecting cultural or policy and system level changes in the acceptance and feasibility of breastfeeding and may be influenced by hospitals increasing “Baby Friendly” maternal care practices and improved lactation support through the Affordable Care Act [40,41]. Strategies to support and promote prolonged breastfeeding are needed, especially given the benefits not only to the child, but the mother as well [42]. 

Some infants under 12 months were already consuming cow’s milk, despite recommendations to avoid such practices. The digestive system of infants is not equipped to handle the solute load from cow’s milk and consumption has been associated with increased risk for dehydration and diminished iron status [23]. Therefore, highlighting the prevalence of early introduction of cow’s milk is important, as it may be contributing to iron inadequacy. Conversely, some children did not consume any milk at all on the day of the survey. A recent analysis by Bailey et al. looked at the nutrient intake in this same group of infants and young children and found that most 12–47.9-month-olds have intakes of vitamin D that put them at risk of inadequacy [22]. Given that our analyses showed that cow’s milk is the number one source of vitamin D in 12–47.9-month-olds, it is concerning that some children are not consuming any milk at all. Moreover, among 24–47.9-month-olds who were consuming milk, nearly 30% were still consuming whole milk. The AAP recommends children switch to low-fat or non-fat milk at age 2; consuming less saturated fat is intended to help reduce the risk of potential long-term health consequences such as heart disease, while still providing the same amount of essential nutrients [11]. In fact, in 2009 the Special Supplemental Nutrition Program for Women, Infants, and Children (WIC) implemented changes to their food package which included a switch to lower fat milk for children 2–4 years, resulting in positive effects on reducing whole milk and saturated fat consumption among young children [43,44,45]. Still, Bailey et al. found that nearly 70% of 24–47.9-month-olds exceeded the saturated fat recommended guidelines in 2016. Given that milks were found to be the number one contributor to saturated fat intake among 12–47.9-month-olds, this is cause for concern. Future interventions should focus on providing clear messaging regarding the importance of milk in the diets of toddlers, while also conveying the need to switch to lower fat varieties after age 2, or at age 1 for those at risk of overweight or obesity. 

Of those children who were consuming milk, amounts were lower among older children (24–47.9 months), which was concurrent with older children consuming higher amounts of 100% juice and SSBs. Our data suggest that not only are some infants being introduced to 100% juice too early in life, but among consumers, they are drinking more than the recommended 4–6 fluid ounces per day [12]. However, it’s important to note that the FITS 2016 preceded recent recommendations to avoid juice before 12 months of age. Among children >12 months old consuming juice, amounts were nearly double the recommended amount at over 8 fluid ounces. Consuming 100% juice at a young age can influence a child’s palate to prefer sweet drinks [46], potentially making the transition to SSBs an easier one. Early 100% juice consumption has been linked to higher 100% juice and SSB intake during early and mid-childhood [19]. Juice and SSBs lack the same satiety signals as consuming whole foods [47,48,49], but provide ample calories; therefore, should be avoided in the first year of life and limited thereafter. While our data show that 100% fruit juice does contribute to intake of some key nutrients, such as calcium (Supplementary Table S2), it is important to consider that even though juice is often considered a healthy beverage, [8] juice does not provide a nutritional advantage over whole fruit due to lack of fiber and relatively high caloric content [12,50]. Nonetheless, it appears that 100% juice consumption, in the general population of 0–47.9-month-olds, may have declined over the past FITS surveys and this warrants further investigation (data not shown) [35]. 

According to our data, 24–47.9-month-olds consumed as many calories from SSBs as from 100% juice, and SSBs were the number one source of added sugar from all foods and beverages for children between 12–47.9 months of age. Recent analyses using 2009–2012 NHANES data show that among children 2–8 years old, the percent of energy from added sugars is 14%, with the primary source being SSBs [51]. Some children may be exceeding the recommended maximum of 10% of calories from added sugars given nearly all 24–47.9-month-olds (90%) from FITS 2016 consumed a dessert, SSB, or sweet snack on the day of the survey [35]. Further analysis is needed to assess the proportion of calories from added sugar since, according to our data, some children were consuming SSBs before 6 months, and among 12–23.9-month-olds nearly a third were consuming them and by 24–47.9 months nearly half, mainly in the form of fruit-flavored drinks. Among consumers of fruit-flavored drinks, intake levels were almost as much as the amount of water being consumed across all age groups. This is concerning given the evidence that links SSB consumption with adverse health outcomes, such as increased risk of weight gain and dental caries and decreased diet quality. Intake of beverages, specifically 100% fruit juice and SSBs, should be monitored throughout early life. Intervention strategies should focus on timely introduction of age-appropriate beverages with an emphasis on appropriate substitutions for calorie dense/nutrient poor beverages, particularity SSBs. For example, substituting water for juice or SSBs is beneficial from calorie standpoint, but replacing milk with water may displace important nutrients. 

Monitoring water intake in young children is important, given their increased risk for dehydration due to greater surface area to volume ratio and higher rates of water loss, compounded by their inability to communicate thirst [52]. Although water is contraindicated before 6 months and should be limited among 6–12-month-olds, older children could benefit from having water at snacks instead of juice or SSBs [7,9]. Our results show that many children are not consuming drinking water as recommended. We found that 10% of 0–5.9-month-olds were consuming water and almost half of 6–11.9-month-olds, taking in an average of 5 fluid ounces per consumer, which does not include water mixed into foods or from foods. Despite water being an important alternative to juice and SSBs among older infants and young children, about 30–38% of all 12–47.9-month-olds did not consume any water on the day of the survey. Studies are limited, but substituting water for SSBs could have positive effects, particularly for children at increased risk for obesity [53,54,55]. Among the few analyses done assessing water intake among children results have been mixed. Drewnowski et al. found among 4–13-year-olds in the U.S., many are not consuming adequate amounts of water [56], where Grimes et al. found the prevalence of inadequate intake of water among infants and toddlers is likely low [14]. More research is needed to assess water intake in young children using consistent methods and classification of water intake, i.e., excluding water consumed as an ingredient. While it is important to factor in the contribution of foods to a child’s water daily water intake, when advising parents on how and when to provide water to their children, it is important to emphasize timely and appropriate portion sizes from beverages.

When looking at beverages consumed at various eating occasions, 24–47.9-month-olds were more likely to consume 100% juice and fruit-flavored drinks at every eating occasion compared to 12–23.9-month-olds, but particularly during snack times. This is concerning for while snacks can provide important nutrients [28], they can also make a significant contribution to overall energy in young children [26,57]. Bleich and Wolfson found that 2–5-year-olds who drink SSBs were more likely to consume salty and sweet snacks, as well as eat more total calories compared to those who do not consume SSBs [58]. Therefore, intervention efforts could focus on snack times as a way to decrease consumption of fruit-flavored drinks and juice, and encourage the consumption of water or milk instead, as well as whole fruit.

As with any rigorous study, there are limitations to consider when interpreting the data. The major limitation is the use of a primary caregiver as a proxy to collect 24-h dietary recalls to measure food intake in children, including if the child was in a child care setting or in the care of another adult. Prior to the dietary interview, respondents were mailed instructions and guidelines for gathering details about foods and beverages consumed during the day including while the child was in child care. Since dietary information is self-reported it is associated with reporting biases and measurement error inherent in dietary recalls. However, this study has many strengths. FITS is a large, nationwide cross-sectional study useful for monitoring intake in young children. Additionally, few reports of beverage patterns extend analyses beyond 2 years; this is the first analysis to use the FITS 2016 data to assess beverage intake from 0 to 47.9 months. Not only do we report the proportion consuming beverages, but also the amounts, calorie and nutrient contributions (including added sugar contributions) and beverages consumed at eating occasions. 

## 5. Conclusions

Overall, the data suggest a misalignment of current beverage patterns with recommendations at these ages. Although improvement was noted when comparing to past survey years, rates of breastfeeding remain low. There continues to be early introduction of inappropriate beverages and excessive intakes of 100% juice and SSBs, particularly fruit-flavored drinks. Some children are not consuming milk, and not enough children are transitioning to low-fat/non-fat milk at 2 years of age. Patterns differ between 12–23.9-month-olds and 24–47.9-month-olds, emphasizing the need for early intervention and guidance. Clearly communicating the recommendations for the introduction and age-appropriate portion sizes of 100% juice and milk to parents and caregivers is important. In addition, future analyses are warranted, including examining how location influences beverage consumption patterns and looking at nutrient intakes in the complementary feeding phase among young children. Understanding current beverage patterns informs future policy, programmatic, and educational interventions along with food industry product improvements and innovations to promote healthy beverage choices and disincentivize unhealthy beverage choices. Conclusions from this study have the potential to inform future dietary guidance such as 2020–2025 DGAs, which will include children under 2 years old for the first time.

## Figures and Tables

**Table 1:** Consumption of beverages by age group: mean percent (±SE) ^1^ consuming, mean grams (±SE) per capita and mean grams (±SE) per consumer among U.S. children, as assessed by 24-h dietary recall and stratified by age group, participating in the Feeding Infants and Toddlers Study, 2016 (*n* = 3235).

	Percent Consuming (±SE)				Grams per Capita (±SE)				Grams per Consumer (±SE)			
	0–5.9 Months	6–11.9 Months	12–23.9 Months	24–47.9 Months	0–5.9 Months	6–11.9 Months	12–23.9 Months	24–47.9 Months	0–5.9 Months	6–11.9 Months	12–23.9 Months	24–47.9 Months
**BABY MILKS**	**98.7 (0.6)**	**93.9 (1.0)**	**16.2 (1.7)**	**4.5 (1.1)**	**512.5 (22.8)**	**311.7 (11)**	**43.5 (4.3)**	**8.6 (2.8)**	**519.1 (22.8)**	**332.1 (11.3)**	**267.9 (17.5)**	**192.2 (49.9)**
Breast milk ^2^	53.5 (2.8)	39.1 (2.0)	11.8 (1.1)	3.5 (0.9)	394.3 (22.0)	221.0 (12.2)	37.9 (4.1)	6.7 (2.3)	736.9 (16.2)	564.7 (11.1)	322.4 (21.8)	192.2 (48.8)
Infant formula	62.2 (2.6)	64.7 (2.0)	4.2 (0.6)	1.1 (0.6)	118.2 (22.8)	89.9 (5.9)	3.9 (1.0)	1.9 (1.4)	189.9 (34.1)	139.0 (8.0)	93.5 (18.6)	173.0 (118.0)
**MILKS ^3^**	**1.9 (0.7)**	**10.7 (1.3)**	**87.1 (1.2)**	**85.8 (2.5)**	**6.9 (4.2)**	**40.5 (7.8)**	**400.6 (11.5)**	**337.0 (16.2)**	**366.5 (139.2)**	**378.8 (55.4)**	**460.1 (11.8)**	**401.1 (16.8)**
Whole milk	1.4 (0.6)	5.8 (1.0)	66.9 (1.8)	26.9 (2.4)	6.5 (4.2)	27.9 (7.2)	312.3 (12.2)	109.2 (13.8)	478.6 (155.5)	482.4 (83.8)	466.8 (14.1)	406.1 (33.3)
Reduced fat milk	0.2 (0.2)	3.5 (0.8)	14.4 (1.3)	33.6 (2.6)	0.2 (0.2)	8.0 (2.2)	50.4 (5.4)	104.0 (10.3)	76.9 (58.3)	226.5 (58.4)	350.4 (20.4)	309.7 (20.9)
Low-fat milk	0.2 (0.2)	1.1 (0.4)	4.7 (0.8)	23.2 (2.2)	0.1 (0.1)	3.1 (2.2)	16.0 (3.5)	93.5 (11.7)	45.8 (0.0)	276.1 (155.8)	337.5 (43.2)	402.6 (29.1)
Non-fat milk	0.1 (0.1)	0.1 (0.1)	2.5 (0.6)	5.6 (1.1)	0.1 (0.1)	0.0 (0.0)	5.2 (1.6)	15.1 (3.0)	214.4 (0.0)	21.4 (1.0)	203.6 (42.3)	272.4 (35.0)
Flavored milk	0.0 (0.0)	0.3 (0.2)	6.1 (0.9)	15.2 (2.1)	0.0 (0.0)	0.5 (0.3)	14.9 (2.5)	38.4 (5.4)	NA	146.1 (47.7)	242.6 (23.6)	252.4 (22.1)
**100% JUICE**	**4.6 (1.0)**	**27.4 (2.0)**	**49.8 (2.0)**	**46.7 (2.7)**	**4.0 (1.3)**	**41.7 (5.1)**	**112.1 (7.3)**	**113.2 (8.6)**	**86.4 (21.4)**	**152.0 (14.9)**	**225.2 (10.8)**	**242.2 (13.1)**
**SSBs ^4^**	**0.9 (0.3)**	**8.5 (1.5)**	**29.1 (1.8)**	**45.5 (2.7)**	**0.9 (0.4)**	**10.5 (2.1)**	**69.0 (6.5)**	**135.9 (10.5)**	**103.8 (30.5)**	**124.1 (13.1)**	**237.0 (14.2)**	**298.7 (16.1)**
Fruit-flavored drinks	0.8 (0.3)	7.3 (1.5)	23.4 (1.7)	34.5 (2.5)	0.8 (0.4)	9.5 (2.1)	55.8 (6.2)	90.7 (7.9)	109 (31.4)	130.8 (14.7)	238.5 (16.5)	263.1 (14.0)
Soft drinks	0.0 (0.0)	0.6 (0.4)	3.1 (0.7)	8.8 (1.5)	0.0 (0.0)	0.2 (0.1)	4.5 (1.5)	19.6 (5.2)	N/A	35.1 (9.3)	144.6 (28.5)	228.8 (40.1)
**WATER ^5^**	**10.0 (1.5)**	**46.2 (2.1)**	**70.0 (1.9)**	**78.1 (2.4)**	**11.4 (2.6)**	**68.5 (5.4)**	**211.2 (8.7)**	**299.2 (15.5)**	**114.8 (22.5)**	**148.5 (9.4)**	**301.8 (9.6)**	**383 (15.9)**

^1^ Standard error. ^2^ Grams of breastmilk consumed via breastfeeding was based on defined amounts by age for infants under 12 months, adjusted for the total volume of other milks consumed during the recall day, or by volume per feeding for children 12 months and older [31]. ^3^ Includes flavored and unflavored cow, goat and plant-based milks; the prevalence of goat and plant-based milks was low and has been reported elsewhere [35], therefore not included. ^4^ Includes fruit flavored drinks, soft drinks and tea and coffee; the prevalence of tea and coffee was low and has been reported elsewhere [35], and therefore not included. ^5^ Includes plain drinking water only.

**Table 2:** Percent (±SE) consuming beverages at meal and snack times among young children participating in FITS 2016 (*n* = 1733).

Eating Occasion	Beverage Type	12–23.9 Months		24–47.9 Months	
		Percent Consuming	SE	Percent Consuming	SE
Breakfast	Milks	41.7%	1.9%	40.8%	2.7%
	Water	17.2%	1.4%	16.9%	2.0%
	100% Juice	15.9%	1.7%	18.7%	2.3%
	Fruit-flavored drink	5.4%	1.0%	7.0%	1.5%
Lunch	Milks	25.2%	1.6%	23.2%	2.1%
	Water	31.0%	1.8%	30.7%	2.6%
	100% Juice	17.6%	1.7%	15.9%	2.0%
	Fruit-flavored drink	8.9%	1.2%	15.9%	2.0%
Dinner	Milks	29.8%	1.8%	31.4%	2.5%
	Water	30.6%	1.8%	31.6%	2.6%
	100% Juice	13.0%	1.4%	13.5%	2.0%
	Fruit-flavored drink	9.0%	1.2%	11.3%	1.5%
Snacks	Milks	56.7%	1.9%	36.4%	2.6%
	Water	50.8%	2.0%	59.0%	2.7%
	100% Juice	24.0%	1.7%	22.1%	2.3%
	Fruit-flavored drink	13.2%	1.4%	16.6%	2.0%

## Supplementary Materials

The following are available online at http://www.mdpi.com/2072-6643/10/7/825/s1, Table S1: Demographic characteristics of infants and young children (0–47.9 months old) participating in the Feeding Infants and Toddlers Study (FITS), 2016 (*n* = 3235), Table S2: Beverage consumption by age group (months): contribution of beverages to percentage of total daily energy intake (%TEI), per capita energy intake and per consumer energy intake from beverages on the day of the survey, FITS 2016 (*n* = 3235), Table S3: The ranking of beverages as sources of energy and nutrients among all foods and beverages consumed on the day of the survey for young children participating in FITS 2016 (*n* = 1733).
