# Supplementary material for: Beverage Consumption Patterns among Infants and Young Children (0–47.9 Months): Data from the Feeding Infants and Toddlers Study, 2016
Source: Nutrients. 2018 Jun 26;10(7):825. doi: 10.3390/nu10070825 (PMC6073729; doi:10.3390/nu10070825)
Supplement: Supplementary file 1 [file nutrients-10-00825-s001.pdf]

SUPPLEMENTARY TABLES

Table S1: Demographic characteristics of infants and young children (0-47.9 months old) participating in the Feeding Infants and Toddlers Study (FITS), 2016 (n=3235).

[redacted]													
		%	*SE*	%	*SE*	%	*SE*	%	*SE*	%	*SE*	%	*SE*
**Child gender**	Male	51	0.88	50	2.04	54	1.66	50	1.49	49	2.86	45	2.90
	Female	49	0.88	50	2.04	46	1.66	50	1.49	51	2.86	55	2.90
**Child's race/ethnicity**	Hispanic	15	0.62	16	1.50	14	1.16	14	1.04	14	1.98	14	2.04
	White (non-Hispanic)	67	0.83	65	1.95	68	1.55	68	1.39	64	2.76	66	2.76
	Black (non-Hispanic)	14	0.61	13	1.36	13	1.11	13	1.01	20	2.28	17	2.19
	Other (non-Hispanic)	4.6	0.37	6.3	1.00	5.0	0.73	4.3	0.61	3.0	0.97	2.7	0.95
**Respondent's education**	Less than high school diploma or GED	4.3	0.36	4.7	0.86	3.9	0.64	3.4	0.54	5.9	1.35	6.5	1.43
	Completed high school or GED	19	0.69	19	1.60	18	1.29	18	1.15	21	2.32	25	2.51
	Some college/postsecondary	23	0.74	27	1.80	24	1.43	23	1.25	18	2.19	17	2.21
	Completed college	38	0.86	37	1.97	38	1.62	41	1.46	36	2.75	36	2.79
	Some graduate work/degree	15	0.63	13	1.36	15	1.19	15	1.06	20	2.28	16	2.14
**Mother's education**	Less than high school diploma or GED	3.9	0.38	4.1	0.89	3.4	0.67	3.4	0.61	5.7	1.53	5.5	1.54
	Completed high school or GED	19	0.77	19	1.77	18	1.43	18	1.28	18	2.53	23	2.85
	Some college/postsecondary	23	0.83	28	2.03	24	1.57	24	1.42	17	2.48	17	2.52
	Completed college	40	0.96	38	2.19	40	1.80	42	1.64	37	3.19	41	3.33
	Some graduate work/degree	14	0.69	11	1.38	15	1.31	13	1.13	23	2.77	14	2.37
**Respondent relationship to child**	Biological/adoptive mother	80	0.71	82	1.56	82	1.29	79	1.20	75	2.48	74	2.55
	Biological/adoptive father	11	0.54	12	1.30	11	1.04	11	0.93	8.2	1.57	10	1.76
	Other	10	0.52	6.2	0.98	7.4	0.87	10	0.88	17	2.14	16	2.11
**Respondent's current marital status**	Married	70	0.81	71	1.86	72	1.50	71	1.36	66	2.75	64	2.80
	Separated or divorced	5.8	0.41	4.9	0.88	3.5	0.61	6.5	0.74	9.0	1.66	8.5	1.63
	Widowed	0.8	0.16	0.7	0.33	0.1	0.11	0.5	0.22	2.3	0.87	2.7	0.95
	Never married	11	0.55	9.0	1.17	11	1.05	11	0.92	13	1.93	14	2.02
	Living with partner	12	0.58	15	1.44	14	1.14	11	0.94	10	1.76	11	1.82
**Household income level**	Less than $10,000	8.5	0.49	9.3	1.19	8.0	0.90	8.2	0.82	11	1.78	7.5	1.53
	$10,000 to $19,999	9.3	0.51	9.3	1.19	9.2	0.96	8.1	0.81	11	1.75	13	1.93
	$20,000 to $34,999	18	0.67	18	1.57	21	1.35	18	1.13	14	1.97	14	1.99

	$35,000 to $49,999	17	0.66	17	1.55	18	1.29	17	1.11	12	1.87	15	2.07
	$50,000 to $74,999	20	0.70	20	1.64	19	1.30	22	1.23	19	2.26	16	2.13
	$75,000 to $99,999	14	0.61	14	1.40	13	1.14	13	1.00	15	2.05	17	2.17
	$100,000 to $149,999	10	0.53	8.3	1.13	8.9	0.95	11	0.91	14	1.99	12	1.91
	$150,000 or more	3.9	0.34	3.7	0.77	2.9	0.56	4.1	0.59	4.3	1.16	6.8	1.46
**Low birth weight (< 2,500 grams)**		8.7	0.50	6.2	0.98	9.0	0.95	8.3	0.82	10	1.71	14	1.99
**Employment**	Respondent worked in last 30 days	50	0.88	44	2.03	47	1.66	52	1.48	55	2.85	59	2.86
	Mother works	47	0.98	37	2.17	43	1.82	49	1.67	57	3.27	60	3.32
	Father works	84	1.97	91	3.39	78	4.22	89	2.84	80	8.00	73	8.07
**Ever breastfed or fed breast milk**		82	0.68	86	1.41	85	1.20	81	1.17	77	2.40	73	2.60
**Anyone in household receiving benefits from SNAP**		26	0.77	24	1.75	26	1.46	26	1.30	30	2.62	29	2.64
**Child attends preschool/daycare etc**		36	0.83	24	1.74	31	1.53	36	1.42	52	2.86	64	2.78
**Child receives benefits from WIC**		36	0.84	41	2.01	42	1.64	34	1.40	29	2.60	25	2.54
**Child's mother receives benefits from WIC**		15	0.63	35	1.95	17	1.25	7.3	0.77	4.6	1.21	5.8	1.36
**Child is mother's first born**		34	0.93	36	2.16	37	1.79	35	1.59	22	2.73	25	2.96

Table S2: Beverage consumption by age group (months): contribution of beverages to percentage of total daily energy intake (%TEI), per capita energy intake and per consumer energy intake from beverages on the day of the survey, FITS 2016 (n=3235).

	Percent total energy intake (±SE)				kcal per capita (±SE)				kcal per consumer (±SE)			
	**0-5.9 months**	**6-11.9 months**	**12-23.9 months**	**24-47.9 months**	**0-5.9 months**	**6-11.9 months**	**12-23.9 months**	**24-47.9 months**	**0-5.9 months**	**6-11.9 months**	**12-23.9 months**	**24-47.9 months**
Baby milks[1]	93 (10)	58 (10)	4 (4)	1 (2)	604 (10)	500 (10)	43 (5)	8 (2)	612 (9)	533 (9)	268 (19)	174 (43)
Breast milk	42 (15)	18 (9)	2 (3)	0 (2)	276 (15)	155 (9)	27 (3)	5 (2)	516 (11)	395 (8)	226 (15)	135 (34)
Infant formula	51 (19)	40 (14)	1 (2)	0 (2)	328 (19)	344 (14)	12 (2)	3 (2)	528 (18)	531 (14)	284 (31)	268 (134)
Milks[2]	1 (3)	2 (4)	18 (7)	11 (9)	5 (3)	24 (5)	237 (7)	176 (9)	243 (87)	228 (33)	272 (7)	210 (10)
Whole cow's milk	1 (3)	2 (4)	15 (7)	4 (8)	4 (3)	17 (4)	191 (7)	68 (9)	323 (95)	294 (51)	286 (9)	252 (21)
Reduced fat cow's milk	0	0	2 (3)	3 (5)	0 (0)	6 (1)	30 (3)	56 (5)	38 (29)	161 (33)	208 (13)	166 (11)
Low-fat cow's milk	0	0	1 (1)	2 (5)	0 (0)	1 (1)	7 (1)	41(5)	19 (0)	116 (65)	146 (17)	179 (13)
non-fat cow's milk	0	0	0	0	0 (0)	0	2 (1)	6 (1)	72 (0)	7 (0)	82 (16)	110 (13)
Flavored cow's milk	0	0	1 (2)	2 (4)	0	0	8 (2)	24 (4)	NA	53 (32)	133 (20)	160 (15)
100% Juice	0	1 (2)	4 (3)	4 (4)	2 (1)	19 (2)	52 (3)	53 (4)	43 (11)	70 (7)	104 (5)	113 (7)
SSBs[3]	0	1 (1)	3 (3)	4 (4)	0 (0)	5 (1)	29 (3)	53 (4)	42 (13)	56 (7)	100 (7)	116 (6)
Fruit flavored drinks	0	1 (1)	2 (3)	3 (4)	0 (0)	4 (1)	24 (3)	39 (4)	47 (13)	60 (8)	104 (7)	112 (7)
Soft drinks	0	0	0	1 (2)	0	0 (0)	2 (1)	8 (2)	NA	14 (4)	58 (12)	85 (17)

[1]For 12-23.9 and 24-47.9 month olds, the percent total energy intake from baby milks includes 0% (3) and 0% (0) from toddler milk, respectively.

[2]Includes flavored and unflavored cow, goat and plant-based milks

[3]Includes fruit flavored drinks, soft drinks and sweetened tea and coffee

Table S3. The ranking of beverages as sources of energy and nutrients among all foods and beverages consumed on the day of the survey for young children participating in FITS 2016 (n=1733).

[illegible]																	
**Milk**[3]	**12-23.9m**	1	18	1	36	NA	NA	1	49	1	69	2	12	1	30	1	22
	**24-47.9m**	1	11	1	19	NA	NA	1	39	1	61	4	7	1	27	1	16
**100% Juice**	**12-23.9m**	8	4	NA	NA	NA	NA	8	2	NA	NA	16	2	NA	NA	NA	NA
	**24-47.9m**	10	4	NA	NA	NA	NA	7	3	NA	NA	13	3	NA	NA	NA	NA
**Sugar-sweetened beverages**[4]	**12-23.9m**	12	3	NA	NA	1	17	NA	NA	NA	NA	NA	NA	NA	NA	NA	NA
	**24-47.9m**	10	4	NA	NA	1	21	NA	NA	NA	NA	NA	NA	NA	NA	NA	NA

[1]These values represent the rank among all foods and beverages that were consumed on the day of the survey. Values presented here only include beverages that ranked in the top 20 foods and beverages that contributed >90% in total of energy intakes in children 12-47.9 months on the day of the survey.

[2]These values represent the percent of total intake of the nutrient listed on the day of the survey that the beverage contributed.

[3]Includes flavored and unflavored cow, goat and plant-based milks

[4]Includes fruit flavored drinks, soft drinks and tea and coffee
